# Contraceptive methods among families at risk for Early Onset Dementia: Related Factors in a Colombian Population

**DOI:** 10.1002/alz.095773

**Published:** 2025-01-09

**Authors:** Juan Camilo Becerra‐Mateus, Daniel Vasquez, Maria Zuluaga, Sofia Rassi, Manuela Gómez, Juan Felipe Quintero, Angela M Andrade‐Villegas, Lucia Madrigal, David Fernando Aguillón Niño, Francisco Lopera

**Affiliations:** ^1^ Grupo de Neurociencias de Antioquia, Facultad de Medicina, Universidad de Antioquia, Medellín, Antioquia Colombia; ^2^ Grupo de Neurociencias de Antioquia, Facultad de Medicina, Universidad de Antioquia, Medellín Colombia

## Abstract

**Background:**

The Alzheimer’s Disease Colombian kindred is the world’s largest autosomal dominant cohort with Early Onset Familiar Alzheimer’s Disease (EOAD) due to a single genetic variant. These families have been studied for decades, identifying the disease progression from early subclinical stages to late dementia stages. Such cognitive and functional decline impacts the mental and physical well‐being of families and caregivers and to our knowledge, how being part of these families affects reproductive desire and contraception has not been studied. Therefore, we aim to explore if sociodemographic data, exposure to the dementia progression of relatives, and knowledge about EOAD is associated with reproductive desire and contraceptive practices in the Colombian kindred.

**Method:**

A cross‐sectional study using a web‐based questionnaire emphasizing socio‐economic status, educational background, relationship with affected relatives, knowledge about EOAD, reproductive health (marital status, number of children, desire to conceive), and contraception (and long‐acting contraceptive methods (LAC)), was sent to cognitively unimpaired individuals from the *PSEN1*‐E280A Colombian kindred. All responders were included (N = 191).

**Result:**

Of all, 58.6% were female, age rank goes from 18 to 47 years, 87% had any contraceptive method, only 29% of the participants had LACs, 2.6% of the participants would like to have children even if they were *PSEN1‐*E280A carriers, and 94% of the participants were interested in being part of genetic counseling programs. The use of LACs was associated with being single and considering that having a family member with EOAD had a negative impact on them. Not wanting to have children or not wanting more children after an unwanted pregnancy was associated with having lived with an EOAD patient, having a higher knowledge of the disease, being single, and considering that having a family member with EOAD had a negative impact on them.

**Conclusion:**

This exploratory study shows that the experience of being part of a family with EOAD can influence sexual and reproductive health. This may be mediated by knowledge about the disease and the experiences of each individual. Further studies on the effect of these factors and possible ways to intervene are needed.

